# Primary Lymphoma of the Colon

**DOI:** 10.4103/1319-3767.56095

**Published:** 2009-10

**Authors:** Leo F. Tauro, Harold W. Furtado, Panambur S. Aithala, Clement S. D'Souza, Celine George, Santhrupth H. Vishnumoorthy

**Affiliations:** Department of General Surgery, Fr. Muller Medical College Hospital, Kankanady, Mangalore, India

**Keywords:** Lymphoma, colon, gastrointestinal, chemotherapy, surgery

## Abstract

Primary lymphoma of the colon is a rare tumor of the gastrointestinal (GI) tract and comprises only 0.2-1.2% of all colonic malignancies. The most common variety of colonic lymphoma is non-Hodgkin's lymphoma (NHL). The GI tract is the most frequently involved site, accounting for 30-40% of all extra nodal lymphomas, approximately 4-20% of which are NHL. The stomach is the most common location of GI lymphomas, followed by the small intestine. Early diagnosis may prevent intestinal perforation; however, the diagnosis is often delayed in most cases. Therapeutic approaches described in two subsets include: Radical tumor resection (hemicolectomy) plus multi-agent chemotherapy (polychemotherapy) in early stage patients, biopsy plus multidrug chemotherapy in advanced stage patients. Radiotherapy is reserved for specific cases; surgery alone can be considered as an adequate treatment for patients with low-grade NHL disease that does not infiltrate beyond the sub mucosa. Although resection plays an important role in the local control of the disease and in preventing bleeding and/or perforation, it rarely eradicates the lymphoma by itself. Those with limited stage disease may enjoy prolonged survival when treated with aggressive chemotherapy.

Primary lymphoma of the colon is a rare tumor of the gastrointestinal (GI) tract and it comprises only 0.2-1.2% of all colonic malignancies.[1,2] The most common variety of colonic lymphoma is non-Hodgkin's lymphoma (NHL). The GI tract is the most frequently involved site, accounting for 30-40% of all extra nodal lymphomas, approximately 4-20% of which are NHL.[2–4] The stomach is the most common location of GI lymphomas; followed by the small intestine.[2,4] We came across two cases of NHL of the colon presenting as a palpable mass and anemia.

## CASE REPORTS

### Case 1

A 45 year-old male patient presented with dull-aching abdominal pain and a lump in the left flank of three months' duration. He showed loss of appetite and weight, and altered bowel habits. A general physical examination revealed that the patient was anemic. Abdominal examination revealed a tender, firm, irregular mass of 9 × 6 cm in the left hypochondrium and left lumbar region with restricted intrinsic mobility and no movement with respiration. Rectal examination did not reveal any abnormalities. His routine blood investigations were normal except hemoglobin which was 8.6g% and ESR which was 46 mm/1^st^ h. Sputum was negative for acid-fast bacilli (AFB). A peripheral smear showed i) microcytic to normocytic, hypochromic RBCs, ii) mild anisopoikilocytosis of RBCs, iii) a total WBC count and cell distribution within the normal range, iv) adequate number of platelets. Stool was negative for occult blood. Liver function tests (LFTs) including levels of liver enzymes, serum bilirubin, serum proteins, and alkaline phospatase were within normal limits. Bone marrow biopsy revealed no abnormalities; a chest X-ray was also normal. Abdominal ultrasonography (USG) detected a hypoechoic mass with pseudo-kidney signs suggestive of a bowel mass noted in the left hypochondrium, measuring about 9 × 6 cm with bowel wall thickening, and a possibility of carcinoma of the left colon. A barium enema study depicted a circumferential growth involving the proximal part of the descending colon, producing a long segment stricture with mucosal irregularity and shouldering, suggestive of malignant growth but with a normal Ileocecal junction. Colonoscopy and biopsy were inconclusive.

The patient was subjected to laparotomy through an infraumbilical midline incision (with supraumbilical extension). This revealed a huge growth at the splenic flexure and proximal descending colon with lymph nodes at the origin of the inferior mesenteric artery. There was no ascitis and detectable secondary deposits in the pelvis, liver, spleen, or peritoneum. Left hemicolectomy was performed with transverse-sigmoid, end-to-end anastomosis. Histopathological investigation revealed diffuse, large cell type, intermediate grade NHL of the left colon. The resected margins were free of tumor but the lymph nodes showed reactive hyperplasia. The postoperative period was uneventful and the patient received six cycles of chemotherapy following surgery (CHOP regimen). The patient was on regular follow-up for two years without any recurrence.

### Case 2

A 60 year-old male patient presented with dull-aching, abdominal pain and a lump in the right lower abdomen of four months' duration. He showed loss of appetite and weight, an evening rise of temperature, and altered bowel habits. He had a cough with hemoptysis for four days. A general physical examination revealed clubbing and anemia. Abdominal examination revealed a tender, soft, irregular, freely mobile mass of 7 × 4 cm size in the right iliac fossa (RIF). Rectal examination results were normal. The patient's routine blood investigations were normal except hemoglobin which was 6.3 g%, and ESR which was 90 mm/1^st^ h. Sputum was negative for AFB and showed no malignant cells. Results of other blood investigations including assays for liver function tests were within normal limits. A peripheral smear revealed microcytic, hypochromic anemia and a chest X-ray showed features of chronic bronchitis. Bone marrow biopsy revealed no abnormality but abdominal USG detected multiple bowel masses with lymphadenopathy in RIF. A barium enema study revealed cecal narrowing, mucosal irregularity, shouldering, and a normal Ileo-cecal junction. USG-guided FNAC of the mass suggested the possibility of lymphoma, which was confirmed by colonoscopy and biopsy.

**Figure 1:**
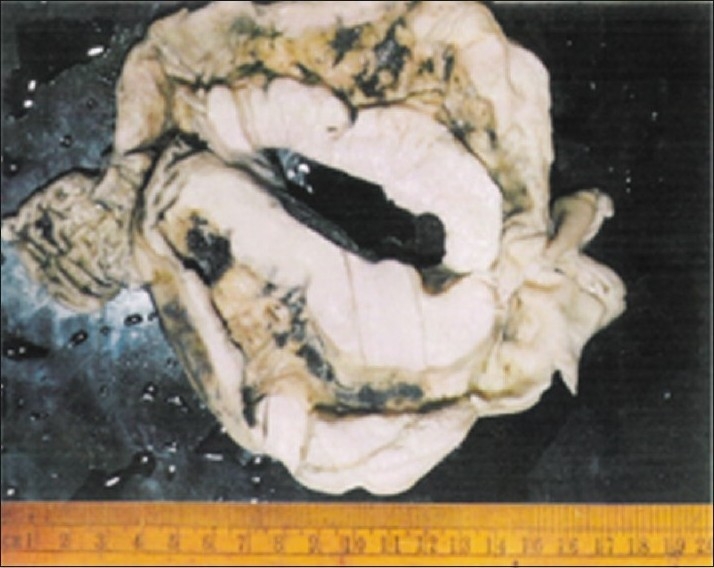
Right hemicolectomy specimen with tumor, lymphoma (cut open)

**Figure 2:**
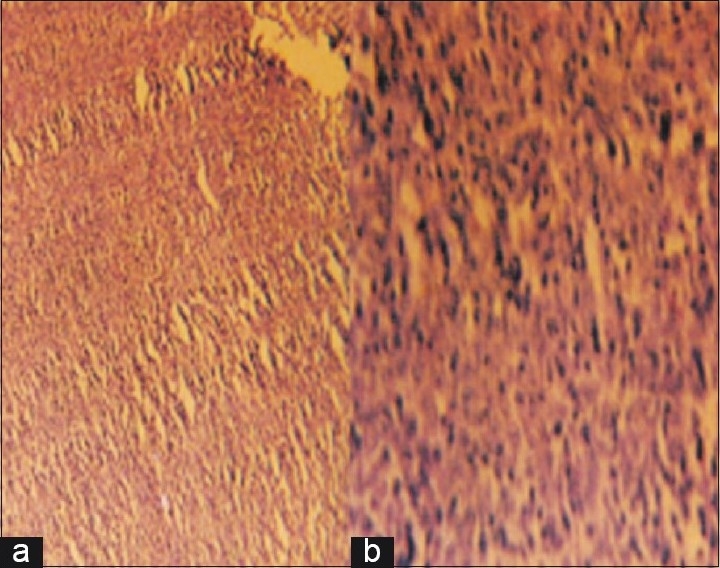
Microscopic picture of NHL (a) under low power (b) Under high power

The patient was subjected to laparotomy through an infraumbilical midline incision which revealed a fleshy growth arising from the cecal wall with lymph nodes in the mesentery. There were no detectable secondary deposits in the liver, spleen, pelvis, or peritoneum. Right hemicolectomy [Figure 1] was performed with ileo-transverse anastomosis. Histopathological investigation revealed diffuse NHL [Figure 2] with no lymph node metastasis and resected margins free of tumor. The patient received six cycles of chemotherapy (CHOP regimen) following surgery. The patient did not show any signs of recurrence at the end of eight months of follow-up.

## DISCUSSION

### Incidence

Lymphomas of the GI tract are the most common type of primary extranodal lymphomas, accounting for 5-10% of all non-Hodgkin's lymphomas. In particular, primary intestinal lymphomas represent about 15-20% of GI lymphomas.[3] Primary lymphoma of the colon is a rare tumor of the gastrointestinal (GI) tract that comprises only 0.2-1.2% of all colonic malignancies.[1,2] GI lymphomas are predominantly located in the stomach (50-60%), whereas intestinal lymphomas are more infrequent and appear in the small bowel (20-30%), the colon, and the rectum (10-20%).[2–5] Colon lymphomas are more frequent in men,[2,5] but are rarely seen in children.[6]

### Pathology

Intestinal lymphomas differ from gastric lymphomas not only in pathology, but also in their clinical features, treatment, and prognosis.[3,7,8] The gross appearance of the tumor may be annular or just a thickened bowel wall. The majority of the colon lymphomas are single (86%),[3,9] but can be multiple or diffuse in nature.[3,10] The term, “primary intestinal lymphoma” includes some particular entities: The western type, immunoproliperative small intestinal disease (IPSID or Mediterranean lymphoma), the enteropathy- associated T-cell lymphoma, and the childhood type. The western type is an uncommon malignancy with a challenging differential diagnosis and an urgent need for therapy. In Western countries, almost all primary colorectal lymphomas are of B-cell lineage whereas they tend to be of T-cell lineage in the East. B-cell lymphomas are more common in older people than are T-cell lymphomas.[3,9,10]

### Clinical features

The most common symptoms of colonic lymphoma are abdominal pain, nausea, vomiting, weight loss, abdominal mass, change in bowel habits, hematochezia,[2,3] obstruction,[2,5] intussusceptions,[11,12] and acute peritonitis due to intestinal perforation.[2,3,9] The lack of specific complaints and the rarity of intestinal obstruction probably accounts for the delay in diagnosis.[2] These bulky masses can usually be palpated by simple physical examination and viewed by ultrasonography.

### Investigations

Abdominal ultrasonography, colonoscopy with sub mucosal biopsy,[2,3] and computed tomography scan of the abdomen are required. Complete blood count, liver function tests, chest X-ray, peripheral smear for hematological studies, and bone marrow biopsy are required to rule out systemic involvement and for staging the disease. Immunohistochemistry may be required in doubtful cases for sub classification.

### Treatment

Combined modality of approach that includes surgical debulking and systemic chemotherapy is the preferred treatment.[13] Different therapeutic approaches were used in two subsets: Radical tumor resection (hemicolectomy) plus multi-agent chemotherapy (polychemotherapy) in early stage patients, biopsy plus multidrug chemotherapy in advanced stage patients.[2,3,10] Polychemotherapy includes CHOP (cyclophosphamide, doxorubicin, vincristin, and prednisolone) or CHOP-like combination chemotherapy or MACOP-B-like regimens.[2,3] Surgery alone can be considered as an adequate treatment for patients with low-grade NHL disease that has not infiltrated beyond the submucosa.[13]

However, it is still thought that the prognosis of intestinal lymphomas is related to surgery; therefore, it seems appropriate and cautious to resect intestinal lymphomas whenever possible.[14,15] Those with limited stage disease may enjoy prolonged survival when treated with aggressive chemotherapy.[2] Radiotherapy is beneficial for incomplete resection or nonresectable disease.

In their study of NHL of the colon, Bairey *et al*,[2] found that 14 patients had primary involvement and three patients had secondary involvement. The ileocecal region and cecum were the most frequent sites of involvement (76%); most patients had bulky disease. Diffuse large cell lymphoma was seen in 11 patients and peripheral T cell lymphoma in one patient. Three patients had mantle cell lymphomas and two had indolent lymphomas: Mucosa-associated lymphoid tissue (*n* = 1) and small lymphocytic (*n* = 1). Eleven patients underwent hemicolectomy: Right-sided in nine and left- sided in two; five diffuse large B-cell lymphoma patients required emergency surgery for intestinal perforation.

## CONCLUSION

Primary colonic lymphomas are rare; the cecum is the most common site of occurrence. Early diagnosis may prevent intestinal perforation; however, the diagnosis is often delayed in most cases. Surgical resection is the mainstay of treatment for localized primary lymphomas, followed by postoperative chemotherapy. Those with limited stage disease may enjoy prolonged survival when treated with aggressive chemotherapy. Surgery alone can be considered as an adequate treatment for patients with low-grade NHL disease that does not infiltrate beyond the sub mucosa. Although resection plays an important role in the local control of the disease and in preventing bleeding and/or perforation, it rarely eradicates the lymphoma by itself.
